# Changes in nano-mechanical properties of human epidermal cornified cells in children with atopic dermatitis

**DOI:** 10.12688/wellcomeopenres.15729.2

**Published:** 2020-07-17

**Authors:** Marek Haftek, Maeve A McAleer, Ivone Jakasa, WH Irwin McLean, Sanja Kezic, Alan D. Irvine

**Affiliations:** 1 Laboratory of Tissue Biology and Therapeutic Engineering, CNRS UMR5305, Lyon, France; 2 Dermatology, Children's Health Ireland at Crumlin, Dublin, Ireland; 3 National Children's Research Centre, Dublin, Ireland; 4 Laboratory for Analytical Chemistry, Dept. of Chemistry and Biochemistry,, University of Zagreb, Zagreb, Croatia; 5 Dermatology and Genetic Medicine, Univsersity of Dundee, Dundee, UK; 6 Coronel Institute of Occupational Health, Amsterdam University Medical Centres, Amsterdam, The Netherlands; 7 Clinical Medicine, Trinity College Dublin, Dublin, Ireland

**Keywords:** Atopic dermatitis, filaggrin, corneocyte stiffness, elastic modulus, Atomic Force Microscopy, Natural Moisturizing Factor

## Abstract

**Background:** Impaired skin barrier is an important etiological factor in atopic dermatitis (AD). The structural protein filaggrin (FLG) plays a major role in maintenance of the competent skin barrier and its deficiency is associated with enhanced susceptibility to mechanical injury. Here we examined biomechanical characteristics of the corneocytes in children with AD and healthy controls.

**Methods: **We recruited 20 children with AD and 7 healthy children. They were genotyped for filaggrin gene ( *FLG*) loss-of-function mutations. Stratum corneum was collected from clinically unaffected skin by adhesive tapes. Cell stiffness (apparent elastic modulus, Ea) was determined by atomic force microscopy and filaggrin degradation products (NMF) by liquid chromatography. Skin barrier function was assessed through trans-epidermal water loss (TEWL) and disease severity by the SCORing Atopic Dermatitis (SCORAD) tool.

**Results: ** Corneocytes collected from AD patients showed a decreased elastic modulus which was strongly correlated with NMF and TEWL, but not with SCORAD. As compared with healthy controls, AD patients had reduced TEWL and NMF levels regardless of *FLG* mutations. NMF was strongly correlated with TEWL.

**Conclusion: **Our findings demonstrate that AD patients have decreased corneocyte stiffness which correlates with reduced levels of filaggrin degradation products, NMF and skin barrier function. Altered mechanical properties of the corneocytes likely contribute to the loss of mechanical integrity of the SC and to reduced skin barrier function in AD.

## Introduction

Atopic dermatitis (AD) is a common inflammatory skin disease, with a lifetime prevalence up to 20% ( Drislane & Irvine, 2020; Weidinger *et al*., 2018). Impaired skin permeability barrier, which is largely provided by the stratum corneum (SC), is an important etiological factor in AD ( Elias & Schmuth, 2009; Kezic *et al*., 2014). SC, the outermost skin layer is comprised of terminally differentiated, anucleated keratinocytes, called corneocytes, embedded in a layered extracellular lipid matrix. Corneocytes are delimited by cornified envelopes (CE) - insoluble 10 nm thick structures composed of highly crosslinked proteins covalently bound to a 5 nm thick monolayer of ceramides replacing cell membranes of viable keratinocytes. They are joined together by modified desmosomes immobilized by cross-linking to the CE and attached to the keratin cytoskeleton filling the cell interior ( Elias, 2008).

Intrinsic or acquired deficiency of an epidermal protein filaggrin is regarded as a major contributor to the compromised skin barrier function in AD ( McAleer & Irvine, 2013; O’Regan *et al*., 2009). Filaggrin has multiple roles in maintenance of the mechanical integrity of the SC barrier. It aligns keratin filaments within the corneocytes and contributes to the composition and structure of CE, thus likely affecting the mechanical properties of the corneocytes. Indirectly, filaggrin influences the SC mechanical integrity through its degradation products, which contribute to a pool of hygroscopic molecules collectively called natural moisturizing factors (NMF). NMF regulate SC hydration, crucial for the plasticity of the skin, its pH and activity of enzymes involved in key homeostatic processes in the SC including lipid synthesis, maturation of CE and desquamation ( Guneri *et al*., 2019; Rawlings, 2014). Therefore, deficiency of filaggrin will likely affect mechanical stability of the SC and its resistance to cracking and chapping, common hallmarks of AD skin.

Recently, we have demonstrated in mouse models that filaggrin and/or NMF deficiency lead to altered topography of the corneocyte surface and to its decreased elastic modulus ( Thyssen *et al*., 2019). In the present study, we investigated whether mechanical properties of the corneocytes are affected in AD developed by humans and how they correlate with *FLG* genotype, NMF levels and permeability barrier function measured by trans-epidermal water loss (TEWL) in clinically non-affected skin of the patients.

## Methods

### Study population

Children with AD ( *n* = 20) were recruited in a dedicated AD clinic in Children’s Health Ireland at Crumlin, Dublin between Jan 2013 and July 2013. Individuals were identified from a cohort of children attending this clinic, sequential patients who attended the clinic and who met the inclusion/exclusion criteria were invited to attend. Severity of disease was determined by a Scoring Atopic Dermatitis (SCORAD) score. All patients were treatment naive, apart from the use of emollients and hydrocortisone 1% cream or ointment. All patients were asked not to use any topical agents for 24 h prior to assessment. As controls, seven children were recruited when attending for elective procedures under general anesthesia. Inclusion criteria for the control group were not having AD, any history suggestive of AD or any other inflammatory skin disease. As this was an explorative study with no prior data to guide us as to effect size, we were not able to calculate power estimates prior to the study commencement. In order to be certain that we fully understood the *FLG* status of participants we only included children with 4 Irish grandparents. Children who had previously used topical corticosteroids stronger than 1% hydrocortisone or who had used topical tacrolimus were excluded. There were no other exclusion criteria. The study was conducted in accordance with the Declaration of Helsinki and was approved by the research ethics committee of Our Lady’s Children’s Hospital, Dublin. Written informed consent was obtained from all patients’ parents.

### Stratum corneum trans-epidermal water loss measurement

TEWL measurements were performed under standardized conditions (room temperature of 22–25 °C and humidity levels of 30–35%). Patients were acclimatized for a minimum of 10 min. prior to measurement. TEWL was assessed on the clinically unaffected skin on the volar forearm using the Tewameter 300 (Courage + Khazaka electronic GmbH, Cologne, Germany).

### Sampling of the stratum corneum by tape stripping

The SC was collected using the previously described method ( McAleer *et al*., 2018; McAleer *et al*., 2019) using circular adhesive tape strips (3·8 cm ^2^, D-Squame; Monaderm, Monaco) and a D-Squame pressure instrument D500 (CuDerm, Dallas, TX, U.S.A.). The adhesive tape was placed on the skin, 2 cm away from the lesion, and pressed for 10 seconds with a pressure of 225 g cm ^2 ^using a D-Squame pressure instrument. Sequentially, eight consecutive tape strips were sampled from the same site and immediately stored at −80 °C. For the NMF analysis the 4 ^th^ tape was used, and for AFM analysis the 7 ^th^ tape.

### Atomic force microscopy (AFM)

**Figure 1.**
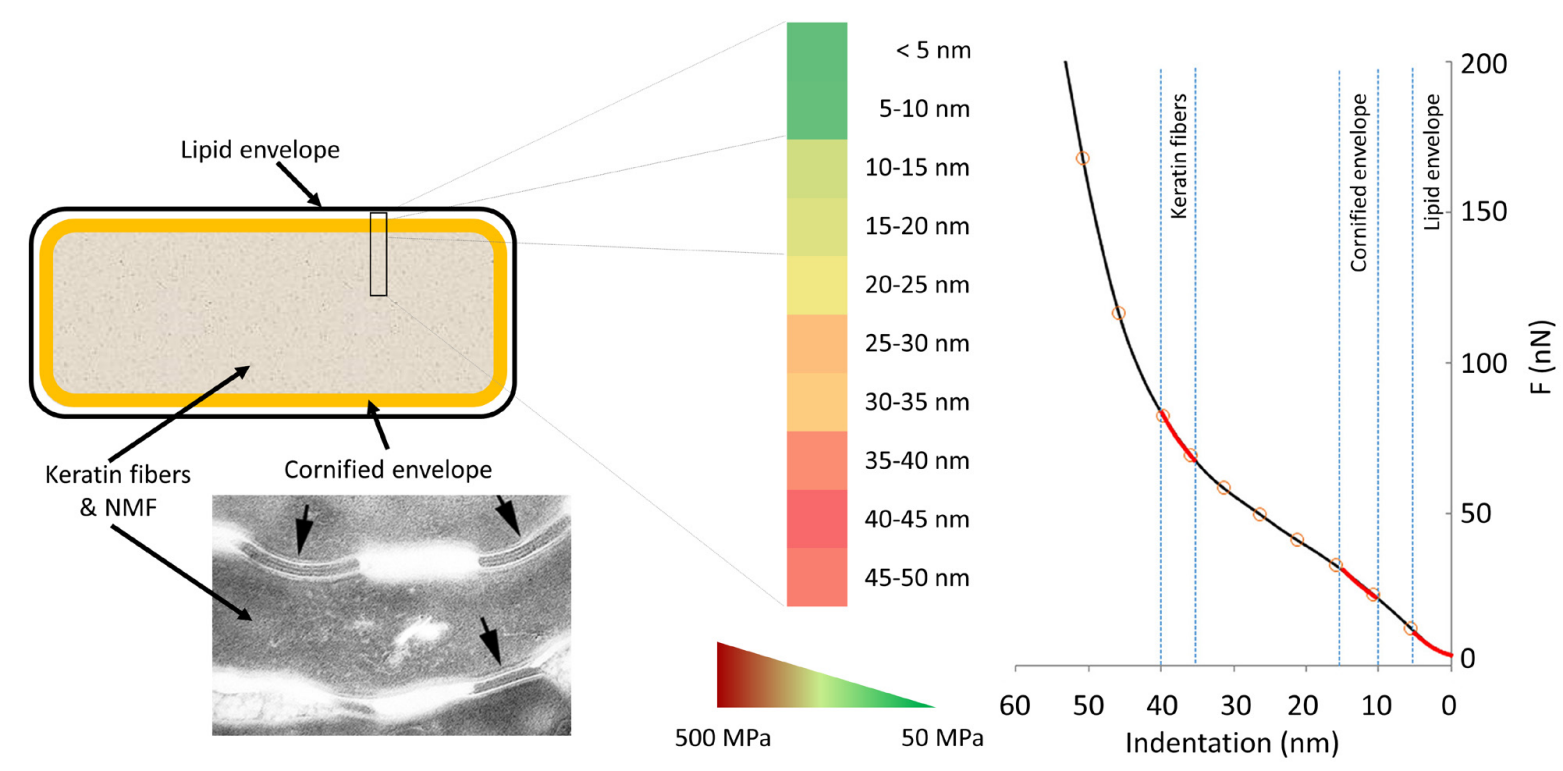
Stiffness tomography study of the mechanical properties (resistance) of a corneocyte (Level L7). The method consists in extracting the elastic modulus at different indentation depths, in intervals of 5 nm, as presented on the force curve (F; in nN, to the right). A representative fitting curve is drawn for three different segments. The surface zone of two 5 nm intervals covers here the lipid envelope. Follow the deeper parts composed of the next two 5 nm intervals representing the cornified envelope and the keratin-rich cell interior recorded at 20 and 50 nm, respectively. A tomographic representation of the obtained stiffness measurements is presented (middle of the panel). The red colour corresponds to high rigidity and the green to a softer material (see the colour scale at the bottom). The heterogeneous mechanical properties can be observed. The lipid envelope is relatively flexible when compared with the corneocyte interior composed of the keratin -rich matrix. The cornified envelope shows an intermediate stiffness. A transmission electron microscopy image of the intermediate part of SC demonstrates the relative narrowness of the lipid and cornified envelopes visualized at the corneocyte periphery, next to the corneodesmosomes (arrows), when compared with the whole cell breadth. (From Milani *et al*., 2018) , copyright held by M Haftek. SC: stratum corneum.

The detailed description of the AFM approach employed for measuring corneocyte stiffness at various cell depths has been recently published ( Milani *et al*., 2018). The experimental setting is schematized in Figure 1. Briefly, tape strips were thawed, and let equilibrate for 30 minutes in the controlled atmosphere of the AFM facility (relative humidity = 45%; temperature = 24°C) and a small piece of each sample was stuck onto glass slides. AFM indentation experiments were carried out with a Catalyst Bioscope (Bruker Nano Surface, Santa Barbara, CA, USA) that was mounted on an optical macroscope (MacroFluo, Leica, Germany) equipped with a x10 objective. A Nanoscope V controller and Nanoscope software versions 8.15 were utilized. All quantitative measurements were performed using standard pyramidal tips (MPP-21100, Bruker AFM probes, Inc.). The tip radius given by the manufacturer was 8–12 nm. Each AFM experiment consisted in acquiring a topographic image of the centre of a cell (10 × 10 µm) and a square matrix of force curves (21–126 readings, mean readings =100; at 1µm steps in the imaged area). For each tape-strip, the study was conducted on 9–10 individual cells (except in case of two AD patients with one *FLG* mutation and one AD patient wild type for *FLG* mutations, 5 and 4 cells were used, respectively) using PeakForce QNM AFM mode at a low frequency (0.5 kHz) with the maximum applied force of 150 nN. Force curves analysis permitted extraction of the quantitative data of the elastic modulus by applying the Hertz–Sneddon model for an indentation ranging from 0 to 50 nm.

### *FLG* genotyping

All patients were screened for the nine most common filaggrin mutations found in the Irish population (R501X, 2282del4, R2447X, S1010X, G1139X, R3419X, 3702delG, Y209X and S3247X), by either restriction digest or direct Sanger sequencing, Full details including primers sequences and PCR cycling conditions are listed in Table 1. PCR amplification reactions were performed on a Biometra T3000 Thermocycler (Göttingen, Germany).

**Table 1.** Screening for *FLG* mutations: PCR, Restriction digest and/or sequencing conditions.

Mutation	Primers	Cycling conditions	Restriction digest or sequencing strategy
501X	F 5’ CAC GGA AAG GCT GGG CTG A 3’ R 5’ ACC TGA GTG TCC AGA CCT ATT 3’	94°C 5min (x1) 94°C 30sec 57°C 30sec 72°C 1min (x35) 72°C 5min (x1)	R501X was screened by restriction digest of a 312bp product R501X introduces a *NlaIII* restriction site. To determine presence of the R501X mutation digest PCR product with *NlaIII*: Run digests on a 3% (w/v) agarose gel. Wildtype allele cuts to give fragments of 182bp, 109bp and 21bp. Mutant allele cuts to give fragments of 127bp, 109bp, 55bp and 21bp.
2282del4	F 5’ AAT AGG TCT GGA CAC TCA GGT 3’ R 5’ GGG AGG ACT CAG ACT GTT T 3’	94°C 5min (x1) 94°C 30sec 57°C 30sec 72°C 1min (x35) 72°C 5min (x1	2282del4was screened by restriction digest of a 811bp product 2282del4 introduces a *DraIII* restriction site. To determine presence of the 2282del4 mutation digest PCR product with *DraIII*: Run digests on a 2% (w/v) agarose gel. Wildtype allele 811bp. Mutant allele cuts to give fragments of 671bp and 140bp
R2447X	F 5’ CCA CAC GTG GCC GGT CAG CA 3’ R 5’ GTC CTG ACC CTC TTG GGA CGT 3’	94°C 5min (x1) 94°C 30sec 64°C 30sec 72°C 1min (x35) 72°C 5min (x1	R2447X was screened by restriction digest of a 185bp product. R2447X introduces a *NlaIII* restriction site. To determine presence of the R2447X mutation digest PCR product with *NlaIII*: Run digests on a 10% TBE polyacrylamide gel. Wildtype allele cuts to give fragments of 95bp, 69bp and 21bp. Mutant allele cuts to give fragments of 69bp, 55bp, 40bp and 21bp.
S1040X	F 5’ CCAGACAATCAGGAACTCC 3’ R 5’ ATGAGTGCTCACCTGGTAGAT 3’	94°C 3 min (x1) 94°C 30sec 62°C 30sec 72°C 1 min (x34) 72°C for 5 min	S1040X was screened by restriction digest of a 375bp PCR product. S1040X creates a *BtsI* site, digestion with this enzyme yields fragments of 251bp and 124bp (mutant allele) whereas the wildtype allele is uncut (375bp).
G1139X	F 5’ CCAGACAATCAGGAACTCC 3 R 5’ ATGAGTGCTCACCTGGTAGAT 3	94°C 3 min 94°C 30sec 62°C 30sec 72°C 1 min (x34) 72°C 5 min	G1139X was screened by restriction digest of a 653bp PCR product. G1139X abolishes a *TspRI* site, digestion with this enzyme yields fragments of 393bp, 157bp, 51bp, 31bp, 15bp and 6bp (wildtype allele) whereas the mutant allele generates fragments of 550bp, 51bp, 31bp, 15bp and 6bp
R3419X	F 5’GCCCATGGGCGGACCAGGA 3’ R 5’GCTTCATGGTGATGCGACCA 3’	94°C 3 min 94°C 30sec 61°C 30sec 72°C 1 min(x34) 72°C 5 min	R3419X was screened by restriction digest of a 332bp PCR product R3419X creates a *NlaIII* site and digestion with this enzyme yields fragments of 307bp, 14bp, 7bp and 4bp (wildtype allele) whereas the mutant allele produces fragments of 252bp, 55bp, 14bp, 7bp and 4bp
3702delG	F 5’ GCA AGC AGA CAA ACT CGT AAG 3’ R 5’CAG ACA ACC TCT CGG AGT CG 3’’	94°C 3 min 94°C 30sec 62°C 30sec 72°C 1 min (x34) 72°C 5 min	3702delG was screened by direct sequencing of a ~217bp PCR product. Use reverse primer as the sequencing primer. Sequenced on Applied Biosystems (Waltham, Massachusetts, USA) 3100 DNA sequencer
Y2092X	F 5’ CA CAG TCA GTG TCA GCA CAG 3’ R 5’ GGC TAA CAC TGG ATC CCC GGG 3’	94°C 3 min 94°C 30sec 62°C 30sec 72°C 1 min (x34) 72°C 5 min	Y2092X was screened by direct sequencing of a 574bp PCR product. Use forward primer a sequencing primer Sequenced on Applied Biosystems (Waltham, Massachusetts, USA) 3100 DNA sequencer
S3247X	F 5’ GTA ATG AGG AAC AAT CAG GAG ACA 3’ R 5’ CTG GGG TGT CTG GAG CCG TGC 3	94°C 5min 94°C 30sec 64°C 30sec 72°C 45sec (x34) 72°C 5min	S3247X was screened by direct amplification of a 268bp product. Use forward primer a sequencing primer Sequenced on Applied Biosystems (Waltham, Massachusetts, USA) 3100 DNA sequencer

### Determination of filaggrin breakdown products in the stratum corneum

Natural moisturizing factor (NMF) component analysis (histidine, pyrrolidone carboxylic acid, *trans*- and *cis*-urocanic acid) and proteins was performed on the fourth consecutive strip according to the method previously described ( McAleer *et al*., 2019). NMF components were extracted with 500 μL 25% (w/w) ammonia solution (Merck Milllipore, The Netherlands, cat. Nr 105432), reconstituted in 500 μL water after evaporating ammonia and analysed by UV high-performance liquid chromatography (HPLC). The column used was a 250 × 3 mm reversed-phase Synergi 4 mm Polar-RP 80A column (Phenomenex, Torrance, CA, USA; catalogue number OOG-4336-Y0) at flow rate of 0.4 mL/min, delivered by Jasco PU-980 HPLC pump (Jasco, Tokyo, Japan). Isocratic elution was performed with a mobile phase, consisting of 4.3 mM hydrochloric acid (Merck Millipore, The Netherlands, catalogue number 6871317), 0.1 mM sodium octane-1-sulfonate (Merck Millipore, catalogue number 1.1830070025), and 2% acetonitrile (Biosolve, The Netherlands, catalogue number 001207802bs). To compensate for variable amount of stratum corneum on the tape, NMF concentrations were normalized for protein concentration. Proteins were determined with a Pierce Micro BCA protein assay kit (Thermo Fischer Scientific, Rockford, IL, USA; catalogue number 687131723235.

### Statistics

Calculations were performed by using Prism 7 software (GraphPad, San Diego, CA). Difference in TEWL, Ea and NMF between AD patients and healthy controls was tested by 2-tailed Student’ or Welch’s *t*-test in the case of non-equal variance. Differences in Ea and NMF between AD patients with and without *FLG* mutations and the healthy controls wild-type for *FLG* mutations were tested by 2-tailed Student’ and Welch’s *t*-test, respectively. Spearman’s rank correlation coefficient was used to test the strength of the relationship between NMF, Ea, SCORAD and TEWL. Distribution of data was tested by Shapiro-Wilks normality test. *P*-value < 0.05 was considered significant.

## Results

### Clinical data

Summary of demographic details of the investigated population is given in Table 2 (individual values are available as underlying data ( Irvine, 2020)). Most of AD children had moderate to severe disease (SCORAD > 25) ( Kunz *et al*., 1997). At least one *FLG* mutation was found to be carried in 15 children, while in the healthy controls there were two heterozygous carriers of *FLG* mutations. The skin barrier function, as assessed by TEWL on non-involved skin of the volar forearm, was significantly lower in AD patients when compared to healthy controls ( *P*<0.01).

**Table 2.** Demographic details of the study participants.

	Patients ( *n* = 20)	Controls ( *n* = 7)
**Gender** ^a^	12 males, 6 females	5 males, 2 females
**Age** (months) (mean, range)	16.8 (3-92)	8.5 (3-12)
**SCORAD** (Average, range)	47.2 (9.7-70.5)	n.a.
**TEWL** (Average, range) ^b, *^	24.7 (9.4-43.1)	9.6 (4.0-13.1)
***FLG* status:** Wild-type Heterozygous LOF mutation Homozygous/compound heterozygous LOF mutations	5 10 5	5 2 0

^a^for two AD patients no gender was noted ^b^ for one healthy control no TEWL was noted *Difference between patients and healthy controls, *P*<0.01; n.a.: not applicable TEWL: Transepidermal Water Loss SCORAD: (SCORing Atopic Dermatitis) tool LOF: Loss-of-function *FLG*: filaggrin gene

### Cell topography

**Figure 2.**
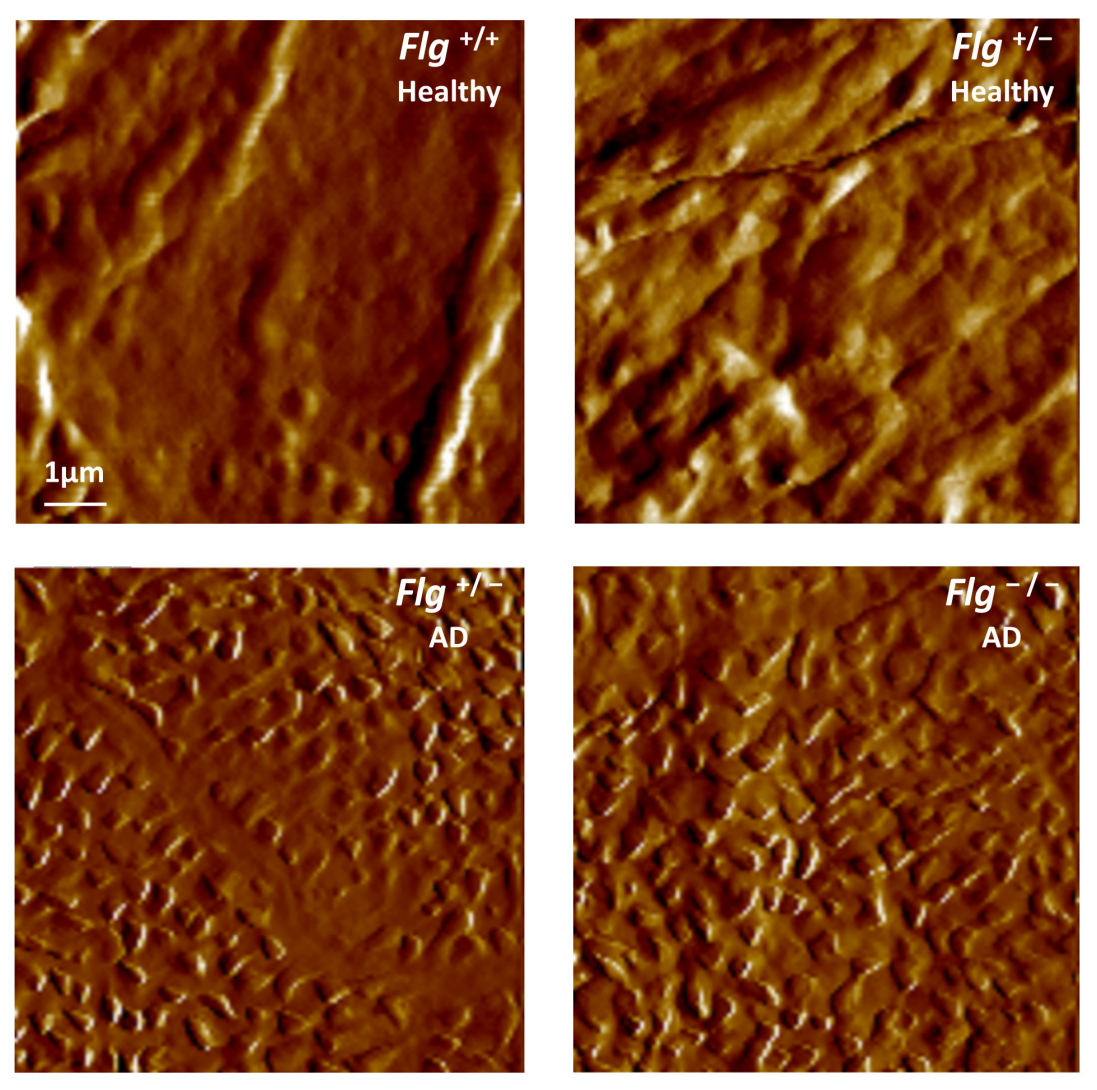
Representative examples of morphological modifications of corneocyte surface in AD compared to healthy controls. Cell surface topography of cells harvested with the 7th consecutive tape strip in healthy subjects and AD patients presenting various *FLG* genotypes. Corneocytes from AD patients show numerous micro-protrusions uniformly distributed at the cell surface.

Corneocytes obtained with the seventh tape strip from the non-involved forearm skin were examined and their lower surface was recorded using AFM topography mode. Representative AFM images regarding disease status and *FLG* genotype are shown in Figure 2 (individual images are provided as underlying data ( Irvine, 2020)). Clear-cut differences in cell surface morphology characterised by presence of numerous micro-protrusions were observed in AD patients, compared to healthy subjects. The slight variation between images recorded within the healthy and AD groups was also noted, according to the *FLG* genotype, but these differences were not quantified in the present study. Of note, cells from subjects that are heterozygous carriers of *FLG*-loss of function (LOF) mutations but did not develop AD, remained free of the surface alteration observed in AD.

### Statistical comparisons between AD and healthy groups with regard to the barrier function, corneocyte stiffness and NMF contents

**Figure 3.**
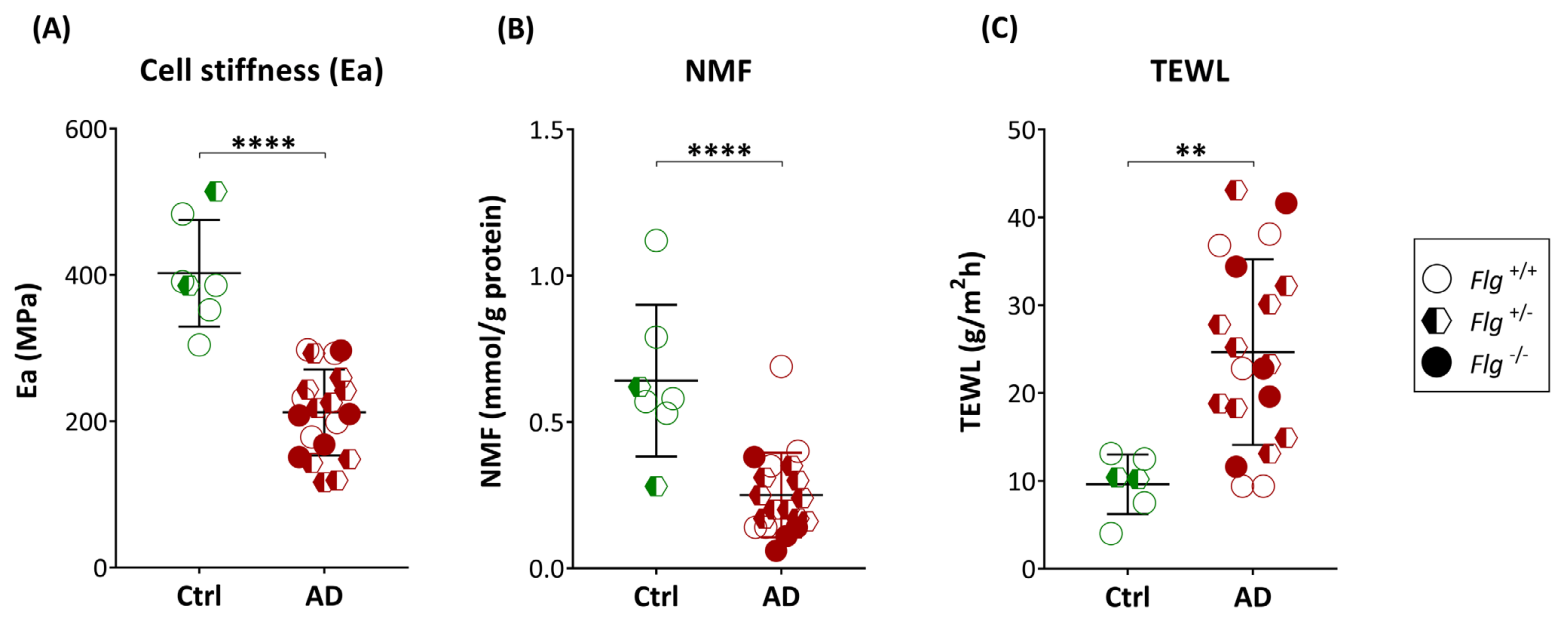
Elastic modulus (Ea) ( **A**); natural moisturizing factors (NMF) ( **B**) and transepidermal water loss (TEWL) ( **C**) in healthy controls and patients. In the figures A and B, *FLG* genotype is given for each subject using different symbols, as indicated in panel A. Differences in Ea, NMF and TEWL between AD patients and controls were tested by a 2-way Student t-test. ** *P*<0.01, **** *P*<0.0001.

As shown in Figure 3A, AD patients had significantly lower elastic moduli (Ea) of corneocytes as compared to healthy controls. A similar pattern has been observed for NMF ( Figure 3B). The values of Ea in AD patients were significantly lower as compared to the healthy controls, irrespective of the presence of *FLG* mutations (P<0.01 and P<0.0001, for the non-carriers and *FLG* mutations carriers, respectively). The same pattern has been observed for NMF (P<0.05 and P<0.01, for the non-carriers and *FLG* mutations carriers, respectively). AD patients had reduced skin barrier function as assessed by TEWL ( Figure 3C).

**Figure 4.**
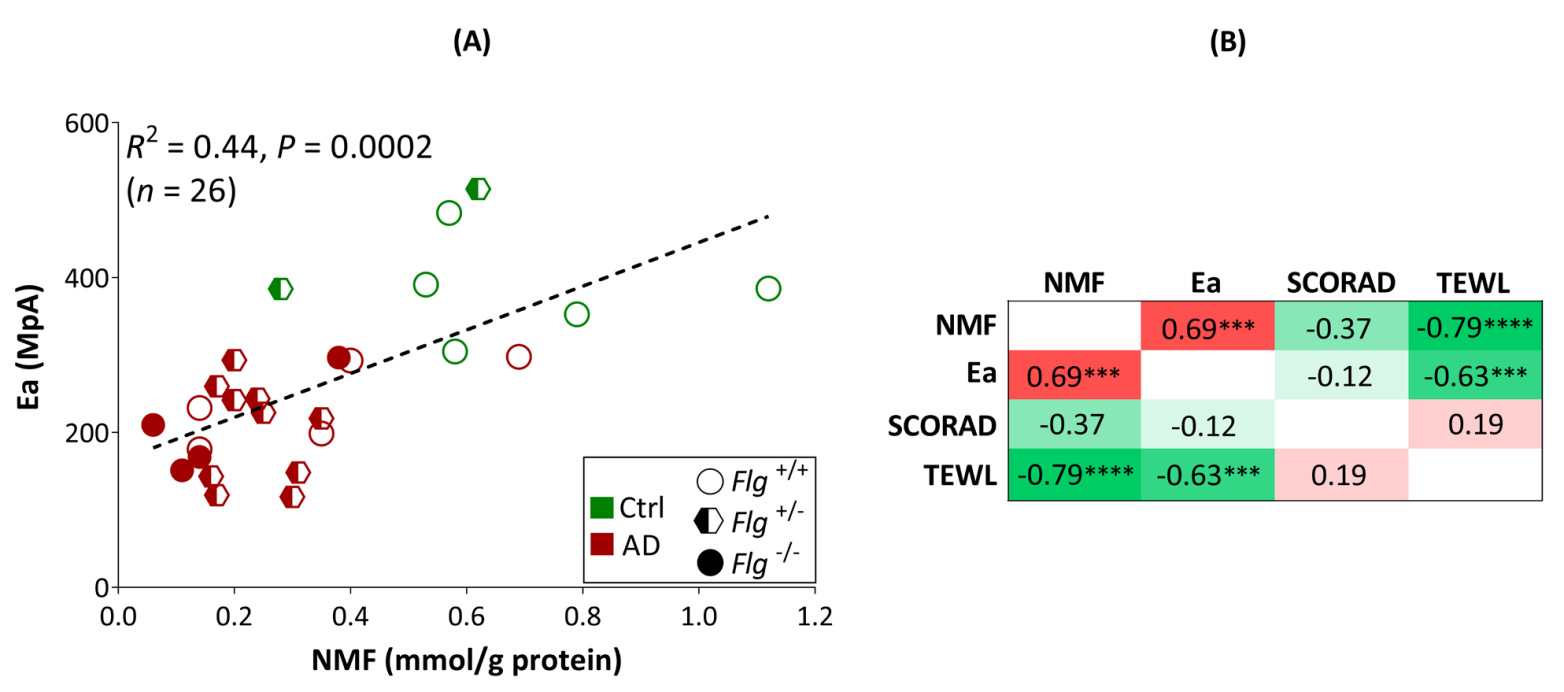
Relationships between investigated parameters. **A**) Regression analysis between Young elastic modulus (Ea) and natural moisturizing factors (NMF). *R* ^2^: determination coefficient. **B**) Heat-map showing relationships between NMF, Ea, SCORAD and TEWL and corresponding levels of significance (P: Spearman correlation coefficient). *** *P*<0.001; **** *P*<0.0001.

The values of apparent elastic moduli (Ea) were strongly and positively correlated with NMF and negatively with TEWL ( Figure 4). In contrast, there was no significant association of Ea with disease severity as assessed by SCORAD. The values of TEWL were negatively correlated with NMF, indicating higher water permeability of SC presenting lower water holding capacity. However, there was no significant association between TEWL and SCORAD ( Figure 4).

## Discussion

According to the “brick and mortar” concept of the SC permeability barrier, cellular elements sealed by intercellular lipids constitute a largely hydrophobic “wall” responsible for the protective functions of the outermost epidermal layer ( Elias, 1983). In AD, these structural elements are or become deficient, leading to an increased transepidermal penetration of environmental antigens and development of immune inflammatory responses ( Elias *et al*., 2008; Elias & Schmuth, 2009).

In a substantial proportion of AD patients, especially those of the North European origin, loss-of-function mutations in the filaggrin gene ( *FLG*) have been incriminated in the pathogenesis of AD ( Drislane & Irvine, 2020; O'Regan *et al*., 2009). Homozygous occurrence of such mutations additionally leads to hyperkeratosis, presenting clinically in the form of Ichthyosis vulgaris ( Thyssen *et al*., 2013), what may be an attempt to compensate for the leaky barrier. The exact mechanism through which filaggrin deficiency leads to insufficient barrier function is not yet fully understood, but there are several lines of evidence demonstrating that not only filaggrin but also its degradation products play a role ( Thyssen & Kezic, 2014). Filaggrin participates in compaction of the keratins within corneocytes, and is the principal source of NMF which is indispensable for correct SC hydration and plasticity, and becomes integrated into the cornified envelopes – the structural scaffolds for organisation of the intercellular lipid layers. We hypothesised that physical proprieties of corneocytes in AD patients would be altered contributing to impaired skin barrier. Indeed, in the present study, we showed that AD patients had significantly softer corneocytes compared to their healthy counterparts of similar age-range and of the same geographic origin, and that their elastic modulus was inversely associated with TEWL, a marker for disordered skin barrier function. Interestingly, a lower elastic modulus was also found in non-involved skin of AD patients irrespective of the presence of *FLG* mutations. The role of inflammation with paracrine diffusion of pro-inflammatory chemokines, cytokines and histamine should be considered as a potential source of systemic subclinical effects on non-involved skin of AD ( Danso *et al*., 2017; Elias *et al*., 2008; Gschwandtner *et al*., 2013; Sawada *et al*., 2012). Several studies convincingly showed that *FLG* expression is downregulated by local Th2 cytokine milieu in AD ( Howell *et al*., 2007; Kezic *et al*., 2011). A recent report incriminated one of the catalytic enzymes from the NMF production cascade in regulation of the inflammatory response. Specifically, deficient expression of keratinocyte bleomycin hydrolase resulted, in addition to the decreased degradation of filaggrin monomers into free amino acids, in an increased release of pro-inflammatory chemokines that are upregulated in skin of AD patients compared to healthy individuals ( Riise *et al*., 2019). Because *FLG* mutations remain the principal determinant of filaggrin expression and its downstream processing cascade, we quantified the levels of filaggrin breakdown products in the tape-stripped corneocytes. In agreement with previous studies, we found that, in comparison with healthy controls, AD patients had reduced NMF levels even in absence of *FLG* mutations. Furthermore, NMF levels were strongly associated with a reduced elastic modulus, indicating that filaggrin breakdown products contribute to the corneocyte mechanical strength. Consistently, in our recent study carried out in mouse models of filaggrin and/or NMF processing, we showed that NMF was strongly correlated with cell stiffness and affected corneocyte topography ( Thyssen *et al*., 2019). Of note, in the present study there was no inverse correlation of SCORAD with the corneocyte elastic modulus. Also this fits very well with data obtained in mouse models of filaggrin and/or NMF deficiency, where softer SC cells and increased TEWL occurred in the absence of macroscopically observable inflammation ( Thyssen *et al*., 2019). This together strongly suggests that inflammation as such is not required for abrogation of the mechanical properties of the corneocytes.

The AFM images of the corneocyte surface in clinically non-involved skin of AD patients showed presence of numerous protrusions, which is in agreement with our previous investigation ( Riethmuller *et al*., 2015). Notably, in that latter study the protrusions showed a dense distribution, co-existing with surface expression of corneodesmosin, suggesting that weakness of the cornified envelope due to imperfect corneocyte maturation was the reason for altered corneocyte surface texture in AD. Cornified cell envelopes undergo progressive maturation within the SC that results in increase of the cell stiffness ( Michel *et al*., 1988; Milani *et al*., 2018). This process appears to rely largely on hydration-dependent activities of transglutaminase 1 and 12R-lipoxygenase ( Guneri *et al*., 2019). It is, therefore, not surprising that in AD, characterized by low NMF content and, consequently, reduced hydration, expression of mRNA and the corresponding proteins composing CE is decreased ( Trzeciak *et al*., 2017).

Various AFM studies of human SC have been previously performed to examine differences in corneocyte morphology and/or stiffness, mostly according to the anatomic localization of the sampled area and to the cell position within the SC depth ( Fredonnet *et al*., 2014; Milani *et al*., 2018). Based on our previous work and the present study, it may be concluded that the apparent elastic modulus (Ea) represents a valid measure of the SC maturity, with all the functional consequences related to a given status. In line with this, the present report confirms that defective permeability barrier function (increased TEWL) in AD correlates with both low NMF values and relative immaturity of corneocytes.

The strength of the present study is homogeneity of the samples regarding SC depth, body location and of the age and ethnic origin of the study population, factors known to influence skin barrier ( Bensaci *et al*., 2015; Fluhr *et al*., 2014; Liu *et al*., 2018; McAleer *et al*., 2018). The main limitation of the study was the small number of healthy subjects with *FLG* mutations which constitute an interesting subgroup for further studies. The number of intragenic copies within the *FLG* gene is known to influence susceptibility to the disease ( Brown *et al*., 2012). This factor could be one more element helpful in dissecting the studied population into subgroups with different clinical outcomes. Furthermore, our findings do not exclude a possible impact of alterations of the corneocyte surface topography, resulting from CE immaturity, on the organisation of extracellular lipid bi-layers – the principal element of waterproofness of the SC barrier ( Elias, 2008; van Smeden & Bouwstra, 2016). Finally, while we were careful to choose clinically non-involved sites, with no obvious inflammation, for sampling we cannot exclude the possibility that subclinical inflammation could influence corneocyte elasticity.

## Data availability

### Underlying data

Open Science Framework: Wellcome Open Access F1KR00CDE. https://doi.org/10.17605/OSF.IO/N4H7F ( Irvine, 2020)

This project contains the following underlying data:

- DATA File_1 Wellcome open_DATA_06.03.2020.pdf (pdf version of individual demographic details of study participants)
- DATA file_Wellcome open_individual DATA_12.04.2020..xlsx (Excel version of individual demographic details of study participants)
- DATA file_Wellcome open_individual DATA_AFM.xlsx (Excel version of all raw Elastic modulus data)
- Haftek *et al* Feb 2020 Individual-AFM-images_20.02.2020._JPEG.zip (Individual images of AFM images)
- Working file_7_DATA file_1_Wellcome open_individual DATA_06.03.2020._corr file.docx (Word Doc version of individual demographic details of study participants)

Data are available under the terms of the Creative Commons Zero "No rights reserved" data waiver (CC0 1.0 Public domain dedication).
