# Supplementary material for: Changes in nano-mechanical properties of human epidermal cornified cells in children with atopic dermatitis
Source: Wellcome Open Res. 2020 Jul 17;5:97. Originally published 2020 May 19. [Version 2] doi: 10.12688/wellcomeopenres.15729.2 (PMC7477645; doi:10.12688/wellcomeopenres.15729.2)
Supplement: Peer review history

# Peer review: Reviewer response for version 2

**DOI:** 10.21956/wellcomeopenres.17739.r39611

**Published:** 2020-09-07

**Authors:** Matthias Schmuth

**Affiliations:** 1 Department of Dermatology, Venereology and Allergy, Medical University of Innsbruck, Innsbruck, Austria

I have no further comments.

Is the work clearly and accurately presented and does it cite the current literature?

Yes

If applicable, is the statistical analysis and its interpretation appropriate?

Yes

Are all the source data underlying the results available to ensure full reproducibility?

Yes

Is the study design appropriate and is the work technically sound?

Yes

Are the conclusions drawn adequately supported by the results?

Yes

Are sufficient details of methods and analysis provided to allow replication by others?

Partly

Reviewer Expertise:

NA

I confirm that I have read this submission and believe that I have an appropriate level of expertise to confirm that it is of an acceptable scientific standard.

# Peer review: Reviewer response for version 2

**DOI:** 10.21956/wellcomeopenres.17739.r39610

**Published:** 2020-07-28

**Authors:** Ellen van den Bogaard

**Affiliations:** 1 Department of Dermatology, Radboud Institute for Molecular Life Sciences (RIMLS), Radboud University Medical Center, Nijmegen, The Netherlands

I have no further remarks on the paper.

Is the work clearly and accurately presented and does it cite the current literature?

Yes

If applicable, is the statistical analysis and its interpretation appropriate?

Yes

Are all the source data underlying the results available to ensure full reproducibility?

Yes

Is the study design appropriate and is the work technically sound?

Yes

Are the conclusions drawn adequately supported by the results?

Yes

Are sufficient details of methods and analysis provided to allow replication by others?

Yes

Reviewer Expertise:

NA

I confirm that I have read this submission and believe that I have an appropriate level of expertise to confirm that it is of an acceptable scientific standard.

# Peer review: Reviewer response for version 1

**DOI:** 10.21956/wellcomeopenres.17242.r38765

**Published:** 2020-06-08

**Authors:** Ellen van den Bogaard, Jos Smits

**Affiliations:** 1 Department of Dermatology, Radboud Institute for Molecular Life Sciences (RIMLS), Radboud University Medical Center, Nijmegen, The Netherlands; 2 Department of Dermatology, Radboud Institute for Molecular Life Sciences (RIMLS), Radboud University Medical Center, Nijmegen, The Netherlands

This manuscript by Haftek *et al.* describes the changes in nano-mechanical properties found in cornified keratinocytes of children with atopic dermatitis. The authors study the skin of twenty AD patients and seven healthy children. Using adhesive tapes, cell stiffness and filaggrin degradation products named natural moisturizing factors were analyzed. In addition, transepidermal water loss (TEWL) was measured and disease severity was assessed using the SCORAD index. This data was complemented with *FLG* genotyping for the nine most common *FLG* mutations. The authors report that cellular stiffness is significantly lower in AD patients compared to healthy volunteers, which is in line with lowered NMF levels and heightened TEWL values. The manuscript is well written and easy to read. The data is convincingly presented and investigated parameters (such as cell stiffness, NMF component concentration, and epidermal water loss) nicely correlate with each other.

 Main remarks:

1. The authors state that they tape-stripped the volunteers and measured NMF content on the 4 ^th^ strip, and cell topography on the 7 ^th^ strip for each and every volunteer, regardless of the skin type (healthy vs. atopic dermatitis). The authors do not present data or explain their choice to use the 4 ^th^ and 7 ^th^ strip. Healthy skin and AD skin (even non-involved skin) can be distinct based on stratum corneum thickness, adhesiveness, water loss, water content, protein degradation product quantity, plasticity, and more. This could directly influence the efficiency of the tape-stripping procedure, and at which stratum corneum layers the investigated parameters can be most effectively measured. Although the method of tape-stripping is widely accepted and used, and non-lesional skin was studied, the authors should explain that the chosen strips are indeed comparable between patients vs controls.
2. The authors sampled non-lesional skin, at 2 cm distance from the lesional sites. It could well be that the skin at this 2 cm distance is affected by the inflammation at the lesional sites (e.g via diffusion of inflammatory molecules or migration of inflammatory cells). Since the non-lesional skin sites were not investigated by tissue morphology (which is understandable given the pediatric population), the authors conclusions that “inflammation as such is not required for abrogation of the mechanical properties of the corneocytes” may therefore be overstated. Could it be that more distant uninvolved skin (e.g. at a different body location) would render a different study outcome? In line with this comment, were any local disease severity parameters assessed (e.g. SUM score), besides the overall SCORAD, to better define the non-lesional skin?   

Minor remarks:

- Methods section, “Atomic force microscopy (AFM)”: The third sentence repeats itself partly.
- Methods section, “ *FLG* genotyping”: ‘&209xA’ should be ‘Y2092X’

Is the work clearly and accurately presented and does it cite the current literature?

Yes

If applicable, is the statistical analysis and its interpretation appropriate?

Yes

Are all the source data underlying the results available to ensure full reproducibility?

Yes

Is the study design appropriate and is the work technically sound?

Yes

Are the conclusions drawn adequately supported by the results?

Yes

Are sufficient details of methods and analysis provided to allow replication by others?

Yes

Reviewer Expertise:

Experimental dermatology, skin inflammation, skin barrier, keratinocyte biology

We confirm that we have read this submission and believe that we have an appropriate level of expertise to confirm that it is of an acceptable scientific standard, however we have significant reservations, as outlined above.

# Peer review: Reviewer response for version 1

**DOI:** 10.21956/wellcomeopenres.17242.r38766

**Published:** 2020-06-08

**Authors:** Matthias Schmuth

**Affiliations:** 1 Department of Dermatology, Venereology and Allergy, Medical University of Innsbruck, Innsbruck, Austria

This manuscript describes biophysical properties of corneocytes collected by D-squame tape strips from patients with atopic dermatitis (AD) as compared to those harvested from healthy control subjects. The research question is interesting, the study design is clear. Some of the measurements (NMF, AFM) were reported in prior independent investigations in skin samples, i.e. citations Riethmuller et al. 2015; and Thyssen et al. 2019. In the present manuscript, the authors confirm structural corneocyte alterations in AD and they provide additional evidence about decreased corneocyte stiffness calculated as elastic modulus. Decreased corneocyte stiffness positively correlates with levels of natural moisturizing factor (NMF) and negatively correlates with transepidermal water loss (TEWL).

 Specific details as to how the authors can address any criticisms and improve the work:

 Table 1: May be moved to supplements.

 Table 2: Available information on gender should be listed not only as number of males – the footnote may be overlooked, this may be misleading.

 Table 2: The information on age suggests that groups are not comparable (upper limit of range and average age are very different). Thus, the authors should avoid stressing similar age range in the discussion on pages 8 and 9. The mean should be shown rather than the average (or both).

 Methods: Please add a more succinct description of how Ea is determined by stiffness tomography.

 Figure 1: The transmission electron microscopy image shown in this figure depicts multiple corneodesmosomes. The figure legend states the narrowness of lipid cornified envelopes next to corneodesmosomes. To what degree does Ea depend on corneodesmosome density?

 Figure 1: It should be checked, if reproducing this figure (previously published in Milani et al. in 2018, figure 2) is in concordance with journal policies.

 Figure 3: Can the TEWL data in figures 3C be shown to include genotype information (as in figures 3A and B)?

 Results/ Discussion: The increased presence of micro-protrusions should be discussed in more detail. What is the structural and biochemical basis for micro-protrusions? Since the authors state that decreased cornified envelope stiffness in AD may be linked to imperfect corneocyte maturation, data on corneocyte maturation, related enzymes (e.g. transglutaminase-1, 12R-lipoxygenase), and presence of mediators of inflammation (e.g. cytokines) would be helpful, if available (not required).

Is the work clearly and accurately presented and does it cite the current literature?

Yes

If applicable, is the statistical analysis and its interpretation appropriate?

Yes

Are all the source data underlying the results available to ensure full reproducibility?

Yes

Is the study design appropriate and is the work technically sound?

Yes

Are the conclusions drawn adequately supported by the results?

Yes

Are sufficient details of methods and analysis provided to allow replication by others?

Partly

Reviewer Expertise:

Dermatology

I confirm that I have read this submission and believe that I have an appropriate level of expertise to confirm that it is of an acceptable scientific standard, however I have significant reservations, as outlined above.
